# Predictive Value of Peripheral Blood Inflammatory Markers and Nutritional Indices for Survival in Young Patients with Advanced Non-Small Cell Lung Cancer: Construction of a Nomogram

**DOI:** 10.3390/curroncol33070391

**Published:** 2026-07-01

**Authors:** Mei Liu, Yu Li, Yiming Lei, Feng Cao

**Affiliations:** 1 Department of Radiation Oncology, The Fourth Hospital of Hebei Medical University, Shijiazhuang 050011, China; 48601044@hebmu.edu.cn (M.L.); liyu_hebmu@163.com (Y.L.); 2 Department of Thoracic Surgery, The Fourth Hospital of Hebei Medical University, Shijiazhuang 050011, China; leiym126@126.com

**Keywords:** young advanced non-small cell lung cancer, survival, inflammatory markers, nutritional indices, nomogram

## Abstract

Background: This study aimed to investigate the prognostic value of peripheral blood inflammatory markers and nutritional indices for overall survival (OS) in young patients with advanced non-small cell lung cancer (NSCLC) at initial diagnosis. Additionally, we sought to develop a survival prediction model based on combined indicators. Methods: We retrospectively analyzed the clinicopathological characteristics, inflammatory markers, and nutritional indices of young patients with advanced NSCLC initially diagnosed at the Fourth Hospital of Hebei Medical University between January 2013 and March 2025. Univariate and multivariate Cox regression analyses were performed to identify independent prognostic factors for OS. Three prognostic models were constructed: a clinical–inflammation model, a clinical–nutrition model, and a clinical–inflammation–nutrition model. Model performance was evaluated using the area under the receiver operating characteristic curve (AUC), concordance index (C-index), and decision curve analysis (DCA), with TNM staging as the reference. Results: A total of 514 patients were included, with a median follow-up of 56.6 months and a median survival time of 27.2 months. Multivariate analysis identified sex, liver metastasis, gene mutation, targeted therapy, white blood cell count, and serum albumin level as independent prognostic factors for OS. Among the three models, the clinical–inflammation–nutrition model showed the best predictive performance, with 1-, 3-, and 5-year AUCs of 0.788, 0.756, and 0.704, respectively, and a C-index of 0.711 (95% CI: 0.696–0.726). DCA further demonstrated its clinical net benefit. Conclusions: Peripheral blood inflammatory markers and nutritional indices are closely associated with survival in young patients with advanced NSCLC. In this exploratory single-center study, a prognostic model integrating clinicopathological factors, inflammatory markers, and nutritional indices showed better apparent predictive performance than models based on single categories of variables or TNM staging, warranting further validation in independent cohorts.

## Simple Summary

Non-small cell lung cancer (NSCLC) is among the most prevalent malignancies encountered in clinical practice. The treatment and prognostic assessment of young patients with advanced NSCLC remain key areas of clinical research. Accumulating evidence indicates that systemic inflammatory responses and nutritional status are closely associated with survival outcomes in cancer patients. In this study, 514 young patients with advanced NSCLC were included. Among the three exploratory prognostic models developed based on different combinations of variables, the combined clinical–inflammatory–nutritional model demonstrated superior predictive performance. This model has the potential to assist clinicians in developing individualized treatment plans that integrate inflammatory and nutritional markers. It may also help optimize nutritional support and anti-inflammatory interventions, thereby improving patients’ quality of life and survival. It also provides objective information for patients and their families regarding disease status. These findings from this preliminary single-center study could be considered as a reference for policy optimization in cancer care. We therefore recommend that incorporating peripheral blood inflammatory markers and nutritional assessments into the routine diagnostic and therapeutic workflow for young patients with advanced NSCLC warrants further investigation in prospective multi-center studies. This approach has the potential to promote rational allocation of healthcare resources, strengthen the diagnostic and treatment capacity of primary care institutions, and reduce regional disparities in care.

## 1. Introduction

Lung cancer is characterized by high incidence and poor prognosis and remains one of the most challenging malignancies to prevent, control, and treat [1,2,3]. Identification of prognostic factors and implementation of targeted interventions are essential for improving survival and optimizing therapeutic decision-making. Currently, the tumor-node-metastasis (TNM) staging system established by the American Joint Committee on Cancer (AJCC) is the most widely used tool for prognostic assessment [4]. Although the TNM classification is periodically updated on the basis of emerging clinical evidence to better meet clinical needs, its prognostic discriminatory ability remains limited in the highly heterogeneous population of patients with advanced non-small cell lung cancer (NSCLC) and therefore does not fully meet clinical needs.

Inflammation is a hallmark of cancer. Persistent chronic inflammatory stimulation may lead to the accumulation of DNA damage, which in turn promotes the release of inflammatory mediators and chemokines, thereby shaping a tumor microenvironment that facilitates tumor progression and metastasis [5]. Peripheral blood mononuclear cells (PBMCs) are important components of systemic antitumor immunity and may reflect the interaction between host immune surveillance and the tumor microenvironment. Alterations in PBMC subsets have been associated with immune dysfunction, tumor immune escape, disease progression, and clinical outcomes in patients with NSCLC [6]. Increased monocyte-driven inflammatory responses may contribute to an immunosuppressive tumor microenvironment and disease progression. Therefore, peripheral blood-derived inflammatory indices, such as NLR, LMR, SII, and SIRI, may serve as accessible surrogate markers of systemic immune–inflammatory status in advanced NSCLC. In parallel, rapid tumor progression consumes substantial energy and can ultimately lead to malnutrition. Malnutrition is particularly common in patients with advanced cancer and is closely associated with survival and quality of life [7,8,9].

Nutritional status is generally poor in patients with advanced NSCLC; however, younger patients often have better performance status and higher expectations regarding quality of life [10,11]. This study aimed to evaluate the prognostic value of inflammatory markers and nutritional indices at diagnosis in young patients with metastatic NSCLC and to develop a novel prognostic model for precise and individualized risk assessment. Our focus on young adults with non-small cell lung cancer (NSCLC) is motivated by robust evidence from prior studies demonstrating distinct clinicopathological features and prognostic outcomes in this population compared with older patients. The findings may, to a certain extent, provide a theoretical basis and clinical evidence to support decision-making.

## 2. Materials and Methods

### 2.1. Study Population

This retrospective study included 514 patients with newly diagnosed, pathologically confirmed NSCLC treated at the Fourth Hospital of Hebei Medical University between January 2013 and March 2025. The inclusion criteria were as follows: (1) age 18–45 years [12,13]; and (2) TNM stage IV disease at initial diagnosis. The exclusion criteria were as follows: (1) a previous history of malignant tumors; (2) hematologic, immunologic, or infectious diseases; (3) prior antitumor treatment; and (4) incomplete clinical or pathological data. This study was approved by the Ethics Committee of the Fourth Hospital of Hebei Medical University (Ethics Review No. 2024KS111).

### 2.2. Patient Characteristics and Outcomes

Baseline characteristics and clinical data were collected for all 514 patients, including sex, age, medical history, treatment details, and lung cancer-specific information. Follow-up was performed through outpatient visits, readmission records, and telephone interviews. The follow-up cutoff date was 31 October 2025. The primary endpoint was overall survival (OS), defined as the interval from diagnosis to death from any cause or last follow-up.

### 2.3. Inflammatory Markers and Nutritional Indices

(1) The neutrophil-to-lymphocyte ratio (NLR) = neutrophil count/lymphocyte count.
(2) The lymphocyte-to-monocyte ratio (LMR) = lymphocyte count/monocyte count.
(3) The platelet-to-lymphocyte ratio (PLR) = platelet count/lymphocyte count.
(4) The systemic immune–inflammation index (SII) = platelet count × neutrophil count/lymphocyte count.
(5) The systemic inflammation response index (SIRI) = monocyte count × neutrophil count/lymphocyte count.
(6) Body mass index (BMI) = weight (kg)/height (m)^2^.
(7) The prognostic nutritional index (PNI) = albumin (g/L) + 5 × lymphocyte count (10^9^/L).

### 2.4. Model Construction and Validation

Univariate and multivariable Cox proportional hazards regression analyses were performed to identify independent predictors of OS (*p* < 0.05). Clinically relevant pathological factors identified in the multivariable analysis, together with preoperative nutritional indices and inflammatory markers that were significant in the univariate analysis, were entered into multivariable Cox proportional hazards regression models. Based on the multivariable results, nomograms for predicting OS were constructed using R software. Model performance was evaluated using the concordance index (C-index), receiver operating characteristic (ROC) curves, and the area under the ROC curve (AUC). Clinical net benefit was assessed using decision curve analysis (DCA).

### 2.5. Statistical Analysis

Statistical analyses were performed using R version 4.3.2 and SPSS version 27.0. The chi-square test was used to compare categorical variables between groups. Optimal cut-off values for inflammatory and nutritional indicators were determined using maximally selected rank statistics. This method evaluates all possible cut-off points and selects the threshold that provides the maximum separation in survival outcomes between groups. Because outcome-oriented cut-off selection may introduce optimism. Bootstrap-based internal validation was conducted using 1000 resamples to estimate optimism-corrected discrimination and calibration. Differences in overall survival were assessed using Kaplan–Meier survival analysis and the log-rank test. Univariate and multivariable prognostic analyses were performed using Cox regression models. Variables with *p* < 0.05 in univariate Cox regression analyses were considered candidates for multivariable Cox regression models. A two-sided *p* < 0.05 was considered statistically significant.

## 3. Results

### 3.1. Clinical and Pathological Characteristics of the Patients

This study included 514 young patients with newly diagnosed advanced NSCLC. The mean age was 41.0 ± 0.7 years. There were 238 male patients and 276 female patients. Among them, 348 harbored driver gene mutations, whereas 166 either tested negative for driver mutations or did not undergo genetic testing, yielding a mutation rate of 67.7%. The median follow-up duration was 56.6 months. Forty patients were lost to follow-up, resulting in a follow-up rate of 92.2%. The median OS was 27.2 months. Detailed baseline clinical characteristics are presented in Table 1.

### 3.2. Selection of Cutoff Values for Inflammatory and Nutritional Indicators

The surv_cutpoint function in the survminer package in R was used to determine the optimal cutoff values for inflammatory markers and nutritional indices. The cutoff values are shown in Table 2.

### 3.3. Clinicopathological Factors Affecting Survival in Young Patients with Advanced NSCLC

Univariate Cox analysis showed that sex, bone metastasis, liver metastasis, gene mutation, chemotherapy, and targeted therapy were associated with overall survival (OS) in young patients with advanced NSCLC (all *p* < 0.05). Female sex, absence of bone metastasis, absence of liver metastasis, presence of gene mutation, no chemotherapy, and receipt of targeted therapy were associated with better OS. Variables with *p* < 0.05 in the univariate analysis were entered into the multivariable Cox analysis, which identified sex, bone metastasis, liver metastasis, gene mutation, and targeted therapy as independent prognostic factors for OS (all *p* < 0.05). Female sex, absence of bone metastases, absence of liver metastases, presence of gene mutation, and receipt of targeted therapy were independently associated with better OS (Table 3).

### 3.4. Impact of Inflammatory and Nutritional Markers on Survival in Young Patients with Advanced NSCLC

Univariate Cox analysis showed that, among the inflammatory markers, white blood cell count, neutrophil count, monocyte count, lymphocyte count, NLR, LMR, PLR, SII, and SIRI were potential predictors of OS in young patients with advanced NSCLC (all *p* < 0.05). Patients with white blood cell count, neutrophil count, monocyte count, NLR, PLR, SII, and SIRI below their respective cutoff values had better prognoses than those with values above the cutoff values. In contrast, patients with lymphocyte counts and LMR above their respective cutoff values had better prognoses than those with values below the cutoff values. Among the nutritional indicators, hemoglobin, serum albumin, BMI, and PNI were identified as potential predictors of OS in young patients with advanced NSCLC (all *p* < 0.05). Patients with hemoglobin, serum albumin, BMI, and PNI above their respective cutoff values had better outcomes than those with values below the cutoff values (Table 4).

### 3.5. Development of the Prognostic Models

#### 3.5.1. The Construction Process of the Three Models

Variables included in model construction: (1) Clinical–inflammatory model: sex, bone metastasis, liver metastasis, gene mutation, targeted therapy, white blood cell count, neutrophil count, monocyte count, lymphocyte count, NLR, LMR, PLR, SII, and SIRI were entered into a multivariate Cox analysis. The results showed that sex, liver metastasis, gene mutation, targeted therapy, and white blood cell count were independent prognostic factors in young patients with advanced NSCLC. (2) Clinical–nutritional model: sex, bone metastasis, liver metastasis, gene mutation, targeted therapy, hemoglobin, serum albumin, BMI, and PNI were included in a multivariate Cox analysis. The results showed that sex, bone metastasis, liver metastasis, gene mutation, targeted therapy, serum albumin, and BMI were independent prognostic factors in young patients with advanced NSCLC. (3) Clinical–inflammatory–nutritional model: sex, bone metastasis, liver metastasis, gene mutation, targeted therapy, white blood cell count, neutrophil count, monocyte count, lymphocyte count, NLR, LMR, PLR, SII, SIRI, hemoglobin, serum albumin, BMI, and PNI were included in a multivariate Cox analysis. The results showed that sex, liver metastasis, gene mutation, targeted therapy, white blood cell count, LMR, and serum albumin were independent prognostic factors in young patients with advanced NSCLC. The multivariate analyses for the three models are presented in Table 5.

#### 3.5.2. Nomogram Construction

**Figure 1:**
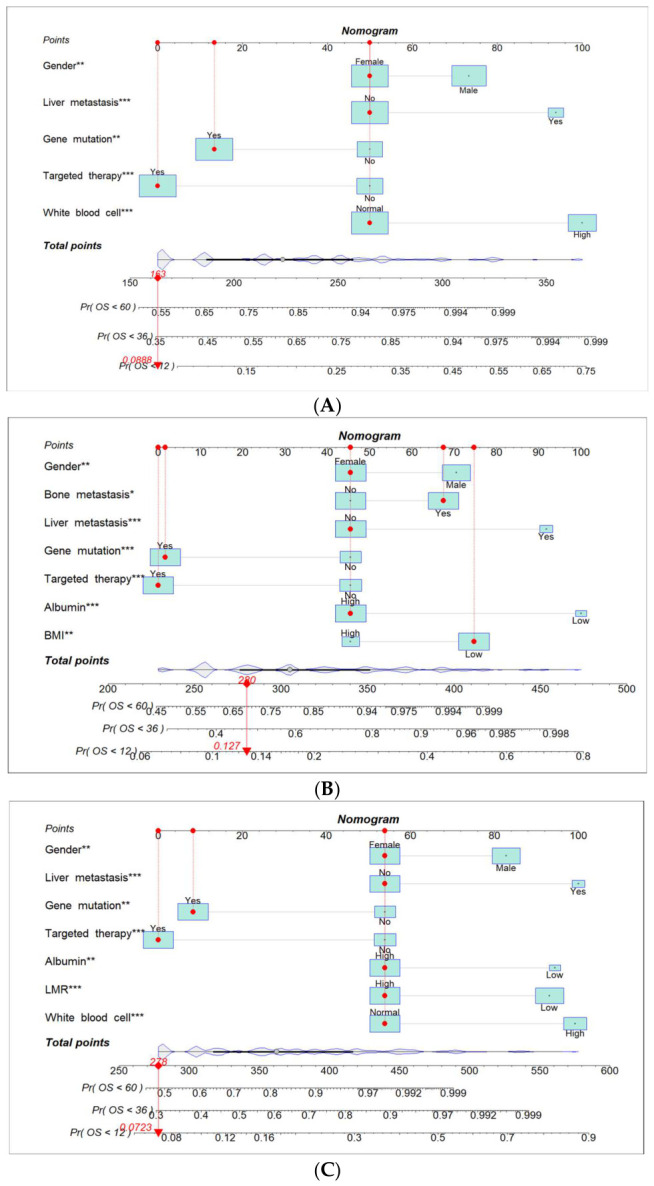
Construction of clinical–inflammation model (**A**), clinical–nutrition model (**B**), and clinical–inflammation–nutrition model (**C**). * *p* < 0.05, ** *p* < 0.01, and *** *p* < 0.001.

Based on the results of the multivariable Cox regression analyses, the independent prognostic factors from the three models were incorporated into nomograms. Each nomogram assigns integer scores (0–100) to individual predictors based on their observed values. The total score-computed as the sum of all predictor-specific points—is transformed via the nomogram’s calibrated regression equation into an estimated 1-year mortality probability for young adults with NSCLC. As shown in Figure 1A, the model integrates five clinically relevant predictors. To derive an individual’s total score, the user aligns each predictor’s value vertically with its corresponding point scale, sums the resulting points, and obtains a composite score ranging from 150 to 350. This total score is then referenced against the bottom probability axis to determine the predicted 1-, 3-, 5-year mortality risk. For example, consider a female patient without liver metastasis, harboring gene mutation, receiving targeted therapy, and exhibiting a normal white blood cell count. The corresponding nomogram points for these five predictors are 50, 50, 13, 0, and 50, respectively, yielding a total score of 163. According to the nomogram, a total score of 163 corresponds to an estimated 8.88% probability of death within 1 year; thus, the predicted 1-year overall survival rate is 91.12%. The results are shown in Figure 1A. In Figure 1B, for example, consider a female patient with bone metastasis, without liver metastasis, harboring gene mutation, receiving targeted therapy, exhibiting a high serum albumin level, and having a low BMI. The corresponding nomogram points for these seven predictors are 45, 68, 45, 2, 0, 45, and 75, respectively, yielding a total score of 280. According to the nomogram, a total score of 280 corresponds to an estimated 12.7% probability of death within 1 year; thus, the predicted 1-year overall survival rate is 87.3%. Similarly, as shown in Figure 1C, consider a female patient without liver metastasis, harboring gene mutation, receiving targeted therapy, exhibiting a high serum albumin level, a high LMR, and a normal white blood cell count. The corresponding nomogram points for these seven predictors are 55, 55, 10, 0, 55, 55, and 48, respectively, yielding a total score of 278. A total score of 278 corresponds to an estimated 7.23% probability of death within 1 year; thus, the predicted 1-year overall survival rate is 92.77%. The same total score may also be aligned with the 3-year and 5-year survival probability axes on the nomogram to estimate medium- and long-term survival outcomes.

Internal validation using 1000 bootstrap resamples showed that the clinical–inflammatory–nutritional model, which incorporated clinical variables, inflammatory markers, and nutritional indices, had superior predictive accuracy (C-index: 0.711, 95% CI: 0.696–0.726) compared with the clinical–inflammatory model (C-index: 0.693, 95% CI: 0.678–0.708), the clinical–nutritional model (C-index: 0.687, 95% CI: 0.672–0.702), and the TNM staging model (C-index: 0.545, 95% CI: 0.527–0.563).

### 3.6. Evaluation of Predictive Models

#### 3.6.1. Discrimination

**Figure 2:**
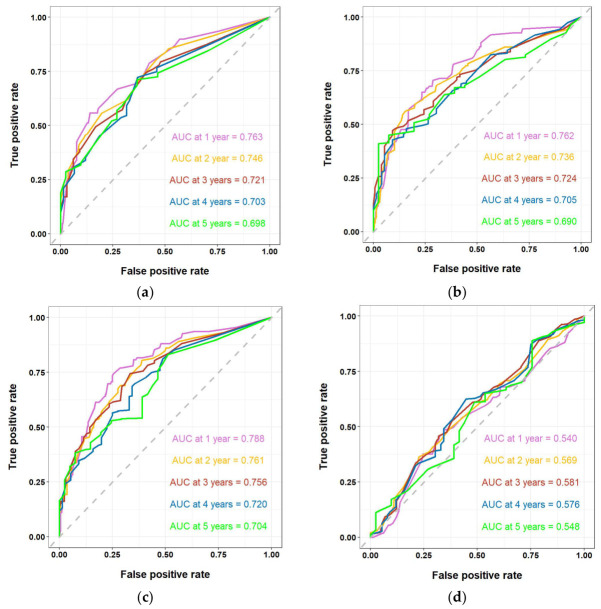
ROC curves for the clinical–inflammation model (**a**), clinical–nutrition model (**b**), clinical–inflammation–nutrition model (**c**), and TNM staging control (**d**).

After 1000 bootstrap resampling, the clinical–inflammatory–nutritional model also demonstrated the best discrimination among all models, with 1-, 2-, 3-, 4-, and 5-year AUC values of 0.788, 0.761, 0.756, 0.720, and 0.704, respectively. These values were higher than those of the clinical–inflammatory model (0.763, 0.746, 0.721, 0.703, and 0.698), the clinical–nutritional model (0.762, 0.736, 0.724, 0.705, and 0.690), and the TNM staging model (0.540, 0.569, 0.581, 0.576, and 0.548); see Figure 2.

#### 3.6.2. Clinical Utility

**Figure 3:**
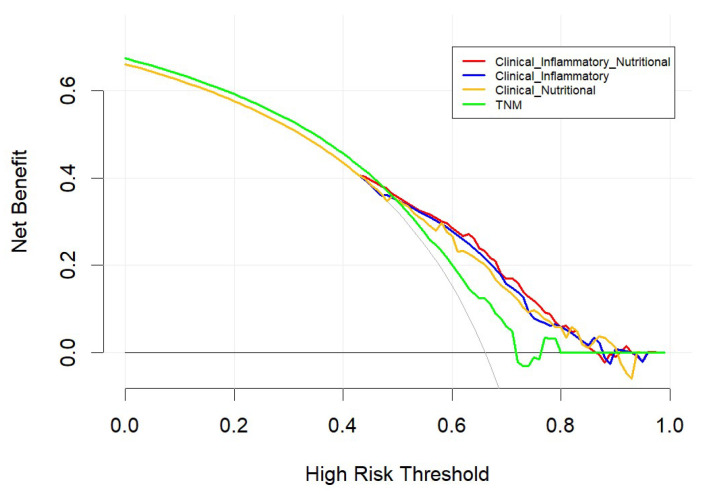
DCA curves comparing the clinical–inflammation model, clinical–nutrition model, clinical–inflammation–nutrition model and TNM staging.

Decision curve analysis showed that the clinical–inflammatory–nutritional model provided greater net clinical benefit across a wider range of threshold probabilities for predicting OS in young patients with newly diagnosed advanced NSCLC (Figure 3).

## 4. Discussion

In this study, we investigated the associations of hematologic inflammatory markers and nutritional indices with OS in 514 young patients with newly diagnosed advanced NSCLC. White blood cell count emerged as an inflammatory marker associated with poor prognosis, whereas serum albumin and body mass index (BMI) were identified as nutritional indicators associated with adverse outcomes. When these factors were integrated with clinically relevant prognostic variables, the resulting comprehensive model demonstrated that the clinical–inflammatory–nutritional model had the best predictive performance. These findings suggest that white blood cell count and serum albumin levels at diagnosis have important prognostic value in young patients with advanced NSCLC.

Leukocytes are a key component of the human immune system. Rather than representing a single cell type, they comprise a diverse family of cells whose major functions include defense against infection, elimination of foreign substances, and maintenance of internal homeostasis [14,15]. To a certain extent, an elevated white blood cell count reflects an enhanced systemic inflammatory state. Excessive release of inflammatory and chemotactic factors may reduce the cytotoxic activity of effector T cells against malignant cells, thereby promoting tumor progression or impairing the response to therapy [16]. Song et al. evaluated 443,540 cancer-free adults from the UK Biobank, including data on total white blood cell counts and their subsets, with follow-up beginning 1 year after baseline. Cox regression was used to estimate hazard ratios for each quartile of white blood cell count or NLR with respect to the incidence of 73 cancer types. Their results showed that elevated white blood cell counts or high NLR may reflect an excessive inflammatory state and thereby promote the development of certain cancers [17]. Wang et al. collected blood samples from 95 patients with small cell lung cancer before chemotherapy and after two treatment cycles. Their study found that the pretreatment circulating tumor cell-to-leukocyte ratio independently predicted worse OS in patients with extensive-stage small cell lung cancer [18]. Currently, studies on the prognostic value of white blood cell count in metastatic NSCLC remain limited. Among the 514 young patients with advanced NSCLC included in this study, the highest white blood cell count was 19.74 × 10^9^/L, and the lowest was 2.97 × 10^9^/L. The cutoff value for white blood cell count was determined using maximally selected rank statistics in the maxstat package in R. The results showed that patients with white blood cell counts > 8.57 × 10^9^/L had a worse prognosis than those with counts below this threshold (*HR* = 1.832; 95% CI: 1.476–2.274; *p* < 0.001). The lymphocyte-to-monocyte ratio (LMR), which reflects the balance between lymphocytes and monocytes, also serves as an indicator of inflammatory and immune status. Previous studies have shown that this ratio is closely associated with prognosis in multiple malignancies. Sekine et al. [19] analyzed clinical data from 87 patients with advanced NSCLC who received nivolumab monotherapy and found that an increase in LMR after treatment was positively associated with objective response rate (ORR) (39.4% vs. 11.8%, *p* = 0.0065). When LMR increased by ≥10%, this change was significantly associated with longer progression-free survival (PFS) (median PFS: 7.3 months vs. 2.5 months, *p* = 0.0049) and longer OS (median OS: 15.6 months vs. 8.9 months, *p* = 0.014). In the present study, patients with an LMR > 3.16 had a better prognosis than those with an LMR ≤ 3.16 (*HR* = 0.497, 95% CI: 0.400–0.617, *p* < 0.001). These findings suggest that, in patients with advanced NSCLC, a low LMR may reflect activation of the inflammatory tumor microenvironment and thus predict poorer survival.

In the combined clinical–inflammation–nutrition model, serum albumin remains an independent prognostic factor, while other nutritional indicators, such as BMI and PNI, do not maintain their independent significance. This might be because albumin not only reflects nutritional reserves but also reflects systemic inflammatory load, liver synthesis function, and metabolic disorders related to cancer. Compared to albumin, BMI is a relatively rough anthropometric measurement indicator and may not accurately reflect cancer-related nutritional depletion. PNI includes albumin and lymphocyte count, and these components may overlap with the inflammatory indicators already included in the model, thereby weakening its independent prognostic effect. Albumin is synthesized by hepatocytes and secreted into the bloodstream, where it is the most abundant plasma protein. Serum albumin is involved in substance transport, immune regulation, and maintenance of vascular endothelial integrity, among other functions [20,21]. In patients with malignant tumors, systemic disturbances and inflammation can suppress albumin synthesis. Although this effect may be less pronounced in early-stage disease, serum albumin levels tend to decline progressively in patients with advanced disease [22,23,24]. Among the 514 patients included in this study, 205 had serum albumin levels below the normal range. Further analysis showed that patients with serum albumin levels > 34.10 g/L had longer OS (*HR* = 0.489, 95% CI: 0.359–0.665, *p* < 0.001). This finding indicates that serum albumin levels at diagnosis are closely associated with OS in advanced NSCLC. These results underscore the importance of closely monitoring nutritional status in patients with cancer and, when necessary, implementing individualized nutritional interventions throughout treatment to improve survival and quality of life.

This study established three exploratory prognostic models. Compared with the clinical–inflammatory model and the clinical–nutritional model, the clinical–inflammatory–nutritional model, which incorporates both inflammatory and nutritional indicators, showed the best predictive performance. Compared with the widely used TNM staging system, the clinical–inflammatory–nutritional model developed in this study provides a more comprehensive and individualized approach to prognostic assessment in young patients with advanced NSCLC and may serve as an important complement to current prognostic tools. The novelty of this study lies in its focus on young patients with newly diagnosed stage IV NSCLC. Rather than evaluating a single inflammatory or nutritional biomarker, this study constructed and compared three progressively integrated models to demonstrate the incremental and complementary prognostic value of inflammatory and nutritional factors beyond conventional clinical variables and TNM staging.

This study has several limitations. First, as a single-center retrospective study, the sample size was limited, which inevitably introduced a degree of selection bias. Moreover, as a single-center study lacks an external validation cohort, it may affect the generalization ability of the model. Second, to obtain a sufficient number of cases for analysis, the study included patients over more than a decade, during which treatment strategies changed substantially and may have introduced heterogeneity. Future studies should incorporate multi-center data to ensure the presence of an external validation cohort. Dynamic monitoring of relevant indicators should also be conducted to achieve more timely and continuous prognostic assessment. In conclusion, this study should be regarded as an exploratory model development study, and further validation is still required before clinical application.

## Figures and Tables

**Table 1:** Patient characteristics.

Variables	N = 514 (%)	Variables	N = 514 (%)
Age		Bone metastasis	
18~40	268 (52.1%)	No	256 (49.8%)
41~45	246 (47.9%)	Yes	258 (50.2%)
Gender		Brain metastasis	
Male	238 (46.3%)	No	351 (68.3%)
Female	276 (53.7%)	Yes	163 (31.7%)
Smoking		Liver metastasis	
No	367 (71.4%)	No	437 (85.0%)
Yes	147 (28.6%)	Yes	77 (15.0%)
Alcohol		Lung metastasis	
No	363 (70.6%)	No	357 (69.5%)
Yes	151 (29.4%)	Yes	157 (30.5%)
Family history		Adrenal metastasis	
No	443 (86.2%)	No	481 (93.6%)
Yes	71 (13.8%)	Yes	33 (6.4%)
Histological type		Gene mutation	
Adenocarcinoma	454 (88.3%)	No	166 (32.3%)
Squamous cell carcinoma	17 (3.3%)	Yes	348 (67.7%)
Other	43 (8.4%)	Chemotherapy	
T stage		No	122 (23.7%)
T1	83 (16.1%)	Yes	392 (76.3%)
T2	132 (25.7%)	Radiotherapy	
T3	77 (15.0%)	No	360 (70.0%)
T4	222 (43.2%)	Yes	154 (30.0%)
N stage		Immunotherapy	
N0	73 (14.2%)	No	458 (89.1%)
N1	60 (11.7%)	Yes	56 (10.9%)
N2	96 (18.7%)	Targeted therapy	
N3	285 (55.4%)	No	179 (34.8%)
M stage		Yes	335 (65.2%)
M1a	198 (38.5%)		
M1b	164 (31.9%)		
M1c	152 (29.6%)		

**Table 2:** Cutoff values for inflammatory and nutritional indicators.

	Variable	Value
Inflammatory indicators	White blood cell count	8.57
	Neutrophil count	5.49
	Monocyte count	0.58
	Lymphocyte count	1.72
	Platelet count	237.00
	NLR	3.21
	PLR	290.80
	LMR	3.16
	SII	1042.68
	SIRI	1.84
Nutritional indicators	Hemoglobin	140.80
	Albumin	34.10
	BMI	26.35
	PNI	46.45

**Table 3:** Univariate and multivariate analyses of clinicopathological variables.

Variables	Univariate Analysis		Multivariate Analysis	
	*HR* (95% *CI*)	*p*	*HR* (95% *CI*)	*p*
Age		0.380		
18~40	1.000			
41~45	0.908 (0.733~1.126)			
Gender		0.042		0.014
Male	1.000		1.000	
Female	0.801 (0.646~0.992)		0.760 (0.611~0.945)	
Smoke		0.557		
No	1.000			
Yes	1.075 (0.845~1.366)			
Alcohol		0.987		
No	1.000			
Yes	0.998 (0.787~1.265)			
Family history		0.672		
No	1.000			
Yes	0.935 (0.685~1.277)			
Histological type		0.520		
Adenocarcinoma	1.000			
Squamous cell carcinoma	1.394 (0.782~2.484)			
Other	0.971 (0.654~1.443)	0.170		
T stage				
T1	1.000			
T2	1.204 (0.850~1.704)			
T3	1.539 (1.050~2.256)			
T4	1.205 (0.872~1.666)			
N stage		0.813		
N0	1.000			
N1	1.221 (0.811~1.840)			
N2	1.119 (0.765~1.636)			
N3	1.088 (0.787~1.504)			
M stage		0.105		
M1a	1.000			
M1b	1.300 (1.014~1.666)			
M1c	1.087 (0.824~1.433)			
Bone metastasis		0.003		0.003
No	1.000		1.000	
Yes	1.391 (1.123~1.724)		1.406 (1.123~1.762)	
Brain metastasis		0.087		
No	1.000			
Yes	1.219 (0.972~1.530)			
Liver metastasis		<0.001		<0.001
No	1.000		1.000	
Yes	1.737 (1.321~2.283)		1.639 (1.227~2.190)	
Lung metastasis		0.937		
No	1.000			
Yes	0.991 (0.782~1.255)			
Gene mutation		<0.001		0.011
No	1.000		1.000	
Yes	0.419 (0.336~0.522)		0.633 (0.444~0.901)	
Chemotherapy		0.004		0.626
No	1.000		1.000	
Yes	1.497 (1.135~1.976)		1.077 (0.800~1.450)	
Radiotherapy		0.320		
No	1.000			
Yes	0.890 (0.707~1.120)			
Immunotherapy		0.816		
No	1.000			
Yes	1.044 (0.728~1.496)			
Targeted therapy		<0.001		0.001
No	1.000		1.000	
Yes	0.424 (0.340~0.528)		0.561 (0.395~0.797)	

**Table 4:** Univariate survival analysis of inflammatory and nutritional indicators.

	Variables	Univariate Analysis	
		*HR* (95% *CI*)	*p*
Inflammatory indicators	White blood cell count	1.832 (1.476~2.274)	<0.001
	Neutrophil count	1.989 (1.600~2.473)	<0.001
	Monocyte count	1.898 (1.523~2.365)	<0.001
	Lymphocyte count	0.767 (0.612~0.961)	0.021
	Platelet count	0.697 (0.548~0.886)	0.003
	NLR	1.824 (1.467~2.269)	<0.001
	LMR	0.497 (0.400~0.617)	<0.001
	PLR	0.671 (0.466~0.966)	0.032
	SII	1.598 (1.288~1.982)	<0.001
	SIRI	1.974 (1.591~2.448)	<0.001
Nutritional indicators	Hemoglobin	0.758 (0.606~0.947)	0.015
	Albumin	0.489 (0.359~0.665)	<0.001
	BMI	0.668 (0.515~0.867)	0.002
	PNI	0.582 (0.467~0.727)	<0.001

**Table 5:** Multivariate Cox survival analyses of models based on clinical, inflammatory, and nutritional indicators in young patients with metastatic NSCLC.

	Variables	Multivariate Analysis	
		*HR* (95% *CI*)	*p*
Clinical–inflammatory model	Gender	0.741 (0.593~0.926)	0.008
	Bone metastasis	1.251 (0.988~1.584)	0.063
	Liver metastasis	1.578 (1.171~2.126)	0.003
	Gene mutation	0.657 (0.473~0.911)	0.012
	Targeted therapy	0.500 (0.355~0.702)	<0.001
	White blood cell count	1.497 (1.071~2.090)	0.018
	Neutrophil count	1.365 (0.906~2.057)	0.137
	Monocyte count	1.135 (0.832~1.548)	0.423
	Lymphocyte count	0.771 (0.566~1.049)	0.097
	NLR	1.210 (0.834~1.755)	0.315
	LMR	0.708 (0.495~1.012)	0.058
	PLR	0.801 (0.539~1.191)	0.273
	SII	0.991 (0.712~1.381)	0.960
	SIRI	0.730 (0.468~1.137)	0.164
Clinical–nutritional model	Gender	0.655 (0.508~0.844)	0.001
	Bone metastasis	1.346 (1.071~1.692)	0.011
	Liver metastasis	1.759 (1.317~2.349)	<0.001
	Gene mutation	0.604 (0.435~0.838)	0.003
	Targeted therapy	0.565 (0.410~0.780)	<0.001
	Hemoglobin	0.805 (0.608~1.066)	0.130
	Albumin	0.580 (0.400~0.840)	0.004
	BMI	0.716 (0.548~0.934)	0.014
	PNI	0.894 (0.678~1.179)	0.427
Clinical–inflammatory–nutritional model	Gender	0.642 (0.494~0.835)	<0.001
	Bone metastasis	1.190 (0.934~1.516)	0.160
	Liver metastasis	1.562 (1.154~2.115)	0.004
	Gene mutation	0.624 (0.445~0.875)	0.006
	Targeted therapy	0.515 (0.366~0.725)	<0.001
	Hemoglobin	0.776 (0.581~1.035)	0.085
	Albumin	0.657 (0.442~0.978)	0.039
	BMI	0.817 (0.622~1.074)	0.148
	PNI	1.206 (0.866~1.680)	0.267
	White blood cell count	1.475 (1.053~2.067)	0.024
	Neutrophil count	1.362 (0.904~2.054)	0.140
	Monocyte count	1.043 (0.757~1.436)	0.799
	Lymphocyte count	0.791 (0.570~1.098)	0.161
	NLR	1.203 (0.827~1.752)	0.334
	LMR	0.678 (0.463~0.992)	0.045
	PLR	0.769 (0.514~1.149)	0.200
	SII	0.978 (0.701~1.365)	0.898
	SIRI	0.713 (0.454~1.120)	0.142

## Data Availability

The raw data supporting the conclusions of this article will be made available by the authors on request.
